# Regulation of BAT thermogenesis via TRPA1-expressing hypothalamic POMC neurons

**DOI:** 10.1080/19768354.2025.2559611

**Published:** 2025-09-29

**Authors:** Arbi Bahtiar Boedi Iman Halanobis, Ju Hwan Yang, Eun-Hye Byeon, Sang Won Park, Hyun Joon Kim, Dawon Kang, Deok-Ryong Kim, Jinsung Yang, Wanil Kim, Dong-Hee Kim, Dong Kun Lee

**Affiliations:** a Department of Physiology, Institute of Medical Science, Gyeongsang National University College of Medicine, Jinju, Republic of Korea; b Department of Pharmacology, Institute of Medical Science, Gyeongsang National University College of Medicine, Jinju, Republic of Korea; c Department of Anatomy, Institute of Medical Science, Gyeongsang National University College of Medicine, Jinju, Republic of Korea; d Department of Biochemistry, Institute of Medical Science, Gyeongsang National University College of Medicine, Jinju, Republic of Korea; e Convergence of Medical Science, Gyeongsang National University College of Medicine, Jinju, Republic of Korea; f Department of Orthopaedic Surgery, Institute of Medical Science, Gyeongsang National University College of Medicine, Jinju, Republic of Korea

**Keywords:** Hypothalamus, energy balance, pro-opiomelanocortin, transient receptor potential ankyrin 1, brown adipose tissue thermogenesis

## Abstract

Pro-opiomelanocortin (POMC) neurons in the hypothalamic arcuate nucleus (ARC) play a pivotal role in regulating brown adipose tissue (BAT) thermogenesis via the sympathetic nervous system. The activation of transient receptor potential ankyrin 1 (TRPA1) has been demonstrated to enhance heat production, particularly in BAT. However, no direct evidence has been reported regarding BAT thermogenesis mediated by TRPA1-regulated ARC POMC neurons. This study aimed to investigate the role of TRPA1-expressing hypothalamic POMC neurons in BAT thermogenesis. To confirm TRPA1 expression in ARC POMC neurons, we employed single-cell reverse transcriptase polymerase chain reaction and immunolabeling techniques. Selective TRPA1 agonists, including capsiate and ASP7663, induced depolarization of ARC POMC neurons, an effect that was inhibited by A967079, a TRPA1-selective antagonist. Furthermore, intracerebroventricular (i.c.v.) administration of ASP7663 increased BAT and core body temperature. The thermogenic effect of ASP7663 in BAT was abolished by co-administration of A967079. Among the BAT thermogenic markers, peroxisome proliferator-activated receptor gamma coactivator 1-alpha and PR domain containing 16 (PRDM16) expressions were considerably upregulated following i.c.v. administration of ASP7663. However, this increase was reversed by A967079, except for PRDM16. These findings indicate that TRPA1-mediated activation of hypothalamic POMC neurons is critical in regulating BAT thermogenesis and promoting energy expenditure.

## 1. Introduction

Energy balance is regulated by anorexigenic pro-opiomelanocortin (POMC) neurons and orexigenic neuropeptide Y (NPY)/agouti-related peptide (AgRP) neurons, both localized in the hypothalamic arcuate nucleus (ARC). These neuronal populations have opposing roles in feeding behavior, with POMC neurons promoting satiety and NPY/AgRP neurons driving food intake (Cansell et al. 2012; Campbell et al. 2017; Friedman 2019; Al-Massadi et al. 2021). The POMC neurons release α-melanocyte-stimulating hormone (α-MSH), a melanocortin receptor ligand that activates melanocortin 3 and 4 receptors (MC3/4R) on neurons in the paraventricular nucleus (PVN), leading to reduced food intake and increased energy expenditure (McMinn et al. 2000; Coll et al. 2004; Timper and Bruning 2017).

Neurons originating from various hypothalamic regions, particularly the PVN, ARC, and lateral hypothalamus, play a critical role in regulating the activity of sympathetic and parasympathetic preganglionic neurons within the autonomic nervous system (Banno et al. 2010; Tang et al. 2020; Li et al. 2023). Several studies suggest that brown adipose tissue (BAT) thermogenesis is closely related to sympathetic nerve activity in the autonomic nervous system (Bartness et al., 2010; Kooijman et al., 2015; Morrison and Madden, 2014 ). Specifically, POMC neurons in the ARC play a pivotal role in suppressing feeding behavior and are also involved in promoting BAT thermogenesis and reducing fat accumulation (Dodd et al., 2015; Tran et al. 2022; Tang et al., 2024).

The transient receptor potential (TRP) ion channel is expressed in nearly all cell types and plays essential roles in a wide range of homeostatic processes (Götz et al. 2017; Bishnoi et al. 2018; Skagen et al. 2023; Zhang et al. 2023). The TRP channels are classified into seven subfamilies based on their amino acid sequence homology, including TRPC (Canonical), TRPV (Vanilloid), TRPM (Melastatin), TRPP (Polycystin), TRPA (Ankyrin), TRPML (Mucolipin), and TRPN (NOMPC) (Fresno et al. 2014; Nilius and Flockerzi 2014; Talavera et al. 2020). Recent studies suggest that the relationship between TRP channels and energy homeostasis, particularly the TRPC subfamily, involves a novel role in regulating energy expenditure in POMC neurons (Qiu et al. 2014; Jeong et al. 2018; Kelly et al. 2018; Vicent et al. 2018). Furthermore, TRPV1 activation by capsaicin and temperature changes has been reported to modulate the activity of POMC neurons by regulating feeding behavior and energy balance (Jeong et al. 2018; Vicent et al. 2018). In addition, oral administration of capsaicin, an agonist of TRP channels, has been reported to activate TRPV1 in sensory neurons of the gastrointestinal tract by inducing β-adrenergic receptor (β-AR)-mediated BAT thermogenesis and promoting the browning of white adipose tissue (Baskaran et al. 2016; Saito et al. 2020; Sun et al. 2021).

TRPA1, also known as ankyrin-like with transmembrane domains protein 1, functions as a sensor for various noxious external stimuli, including extreme cold, pungent odors, reactive chemical species, and endogenous signals associated with cellular stress and damage (Nilius and Flockerzi 2014; Jha et al. 2015; De Logu et al. 2017; Meents et al. 2019; Wang et al. 2024). As a cold-sensing thermoreceptor, TRPA1 plays a crucial role in thermogenesis and is activated by temperature changes, particularly exposure to temperatures < 17°C. In addition, TRPA1 responds to various natural compounds and synthetic agonists, including capsiate, anisaldehyde, cuminaldehyde, and tiglic aldehyde (Schepers and Ringkamp 2010; Zhong et al. 2011). Recent studies suggest that TRPA1 channels may be involved in BAT thermogenesis and lipid metabolism. Furthermore, TRPV1 and TRPA1 share structural similarities by responding to similar pharmacological compounds and enhancing uncoupling protein 1 (UCP1) expression in BAT, thereby facilitating thermogenesis (Oi-Kano et al. 2017). Although these findings indicate a potential role of TRPA1 in BAT thermogenesis, its specific involvement in hypothalamic POMC neurons remains unclear.

Therefore, this study aimed to investigate the role of TRPA1-expressing hypothalamic POMC neurons in BAT thermogenesis. These findings suggest that TRPA1 may play a role in regulating energy homeostasis and obesity.

## 2. Materials and methods

### 2.1. Animals preparation

The study was conducted in accordance with a scientifically evaluated protocol (GLA-100917-M0093) and the National Institutes of Health's guidelines. The mice utilized in the present study were mixed C57BL/6, FVB, and 129 strain backgrounds known as POMC-Cre (stock# 005965, Jackson Laboratory, Bar Harbor, ME, USA) and POMC-eGFP (stock# 009593, Jackson Laboratory). All experiments and animal care procedures were approved by the Gyeongsang National University Institutional Animal Care and Use Committee (GNU IACUC, GNU-200820-M0053).

### 2.2. Single-Cell reverse transcriptase polymerase chain reaction (RT–PCR) and real-Time PCR

Single POMC neurons were isolated via aspiration into a patch pipette from brain slices prepared using the method employed for patch clamp recording. The reverse transcription (RT) reaction to obtain complementary DNA (cDNA) was performed using a REPLI-g WTA single-cell kit (Qiagen, Hilden, Germany). The samples, comprising total RNA from single POMC neurons in a glass pipette, were expelled into a microcentrifuge tube containing 2 μL of lysis buffer (with a total volume of 4.5 μL, including 2.5 μL single-cell sample). The mixture was incubated at 24°C for 5 min and then cooled to 4°C. Subsequently, the samples were incubated with 1 μL gDNA wipeout buffer at 42°C for 10 min. 3.5 μL RT mix (comprising 0.5 μL oligo [dT] primer, 2 μL RT buffer, 0.5 μL random primer, and 0.5 μL RT enzyme mix) was added. The tubes were incubated at 42°C for 1 h, followed by a 3 min incubation at 95°C. Next, the tubes were incubated at 24°C for 30 min with 5 μL ligation mix (consisting of 4 μL ligase buffer and 1 μL ligase mix). The reaction was terminated by incubating at 95°C for 5 min. Following the addition of the amplification mix (14.5 μL buffer and 0.5 μL DNA polymerase), samples were incubated at 30°C for 2 h and at 65°C for 5 min. The RT–PCR amplicon obtained from a single POMC neuron was used for PCR analysis, confirming the expression of TRPA1 mRNAs through 2% agarose gel electrophoresis. The primers used for RT–PCR were the following: F5′- gcaacacatcaaagagctgg-3′ and R5′-atggacacattgaagccaag-3′ for Trpa1 (NM_001348288.1), and F5′-ctcaacacgggaaacctcac-3′ and R5′-ccatccaatcggtagtagcg-3′ for 18S rRNA (NR_003278.3).

### 2.3. Electrophysiological recordings

A vibratome (7000 smz-2; Campden Instruments, Loughborough, UK) was used to create 200 μm thick transverse brain slices. The pipette solution contained 130 mM K-gluconate, 5 mM CaCl_2_, 10 mM EGTA, 10 mM HEPES, 2 mM MgATP, 0.5 mM Na_2_GTP, and 10 mM phosphocreatine. To measure membrane potentials from a brain slice, a piece of tissue was placed in a recording chamber perfused with artificial cerebrospinal fluid (aCSF containing 113 mM NaCl, 3 mM KCl, 1 mM NaH_2_PO_4_, 26 mM NaHCO_3_, 2.5 mM CaCl_2_, 1 mM MgCl_2_, and 5 mM glucose in 95% O_2_/5% CO_2_) at 1.5–2 mL/min. Recording chambers were assembled at the surface of an upright infrared differential interference contrast microscope (Olympus BX51WI; Olympus, San Jose, CA, USA) positioned on a Gibraltar X-Y table. The brain slices were examined using a 40X water immersion objective. Whole-cell current-clamp recordings were performed on visually identifiable ARC POMC neurons in POMC-eGFP mice brain slices at a resting potential of −70 mV. Membrane potentials were measured in the whole-cell format using a MultiClamp 700B amplifier (Molecular Devices, San Jose, California, USA). Electrophysiological data were low-pass filtered at 2–5 kHz, stored on a computer, and analyzed offline with pClamp 11 software (Molecular Devices). Membrane potentials recorded every 30 s were treated as single data points. Paired t-tests were used to compare ten data points before and after medication application. All recordings were done at 30 ± 2°C.

### 2.4. Immunohistochemistry (IHC)

Male mice (5–6 weeks old) were anesthetized with avertin (0.5 g 2,2,2-tribromoethanol and 1 mL 2-methyl-2-butanol in 40 mL total volume, Sigma Aldrich, St. Louis, USA) and transcardially perfused with 10 U/mL heparin in phosphate-buffered saline (PBS). Mouse brains and BAT tissues were immersed in 4% paraformaldehyde in PBS at 4 °C overnight for fixation. The following day, brain and BAT tissues were sectioned at 40 and 80 μm thickness, respectively, using a vibratome (Leica Microsystems, Buffalo Grove, IL, USA), and stored in 1× PBS containing 0.001% sodium azide at 4 °C. For immunofluorescence staining, the slices were rinsed three times in PBS for 10 min each. After blocking with 1 mL of 0.5% TritonX-100/ bovine serum albumin (BSA) for 1 h, the slices underwent three 10-minute PBS washes. The brain slices were then incubated overnight at 4°C on a rolling shaker with primary antibodies (in 2% BSA/PBS), including anti-rabbit TRPA1, anti-mouse beta subunit cholera toxin B (CTB), anti-rabbit c-fos (1:500, Abcam, Danvers, MA, USA), or anti-mouse mcherry antibody (1:500, Novus, Vancouver, BC, Canada). Alexa fluor 594 anti-rabbit secondary antibody (1:1,000, Abcam) with 2% BSA/PBS was stained for 1 h at room temperature following three PBS washes. Neutral lipid staining was performed by incubating tissues with LipidTox Green (1:200 dilution; Thermo Fisher Scientific, H34475) for 30 min at room temperature. Images were captured using an Olympus fluorescent microscope.

### 2.5. Stereotaxic surgery and drugs administration

Mice were anesthetized with avertin (0.5 g 2,2,2-tribromoethanol and 1 mL 2-methyl-2-buthanol in 40 mL total volume, Sigma Aldrich) and positioned in a stereotaxic device. Sterile guide cannulas (RWD, Sugar Land, TX, USA) were implanted into the lateral ventricle (anterior-posterior, + 0.85 mm; medial-lateral, + 0.5 mm; dorsal-ventral, −3.0 mm) for intracerebroventricular (i.c.v.) injection of ASP7663 (1 μL of 10 μM) and A967076 (1 μL of 5 μM, Sigma Aldrich) under sanitary circumstances. Following ≥ 1 week of postoperative recovery, a flexible implantable microprobe (IT-18 or 21, Physitemp Instruments, NJ, USA) was implanted into the interscapular BAT, and a thermoprobe (RET-4, Physitemp Instruments, NJ, USA) was inserted into the rectum.

### 2.6. Measurement of BAT and core body temperature

Core body and interscapular brown adipose tissue (BAT) temperatures were measured under isoflurane anesthesia at room temperature (24 °C). A rectal thermoprobe (RET-4; Physitemp Instruments, NJ, USA) was inserted to monitor core temperature, and a flexible implantable microprobe (IT-18 or IT-21; Physitemp Instruments, NJ, USA) was placed beneath the BAT for local temperature recording. Temperature data were acquired using the THERMES-USB Temperature Data Acquisition System (Physitemp Instruments, NJ, USA) from 2–3 mice per measurement. To maintain thermal stability during anesthesia, animals were placed on warming pads and exposed to heat lamps.

### 2.7. Western blot

BAT tissues were extracted and homogenized in RIPA buffer with protease inhibitors (Thermo Scientific) using a Beadbug microtube homogenizer (Sigma Aldrich) at maximum frequency, with samples kept on ice until well homogenized. The homogenized samples were centrifuged at 6,000 g for 15 min at 4°C. Following centrifugation, a pipette tip was gently pushed through the fat coating to gather only the lysates, which were then transferred to a fresh tube. Triton X-100 was added to a final concentration of 10% and incubated for 1 h at 4°C. Finally, samples were centrifuged twice at 12,000 g for 15 min each at 4°C, and supernatants were stored. Sodium dodecyl sulfate-polyacrylamide gel electrophoresis was performed using a 10% gel to separate the proteins in supernatants (10 μg). A polyvinylidene difluoride membrane (Merck, Darmstadt, Germany) activated by methanol was used to hold the separated proteins. After three 10-minute washes, the membrane was blocked with a solution consisting of 0.1% Tween-20, Tris-buffered saline, and 5% skim milk. Primary antibodies against UCP1 (1:20,000, Abcam), transmembrane monocarboxylate transporter 1 (MCT1) (1:1,000, Thermo Fisher Scientific, Tewksbury, MA, USA), PR/SET Domain 16 (PRDM16) (1:1,000, Abcam), growth differentiation factor-8 (GDF8) (1:1,000, Abcam), and peroxisome proliferator-activated receptor-gamma coactivator 1-alpha (PGC1α) (1:1,000, Abcam) or interferon regulatory factor 4 (IRF-4) (1:1,000, Santacruz Biotechnology, Dallas, TX, USA) were used as protein markers, overnight at 4°C, and rewashed three times, the incubated with horseradish peroxidase-labeled goat anti-rabbit or mouse secondary antiserum (1:1,000) (Thermo Fisher Scientific) for 1 h at room temperature. Immunoreactive protein bands were identified with an iBright Western blot imaging system (Thermo Fisher Scientific) and increased luminescence reagents, Westsave Up (reagent A to B ratio = 1:500; Ab Frontier, Seoul, Republic of Korea). To normalize the blots, the original membrane was stripped and stained with mouse primary antiserum against β-actin (1:5,000) from Sigma Aldrich. The immunoreactive protein bands were semi-quantified using Image J software.

### 2.8. Statistics

Statistical analyses for Western blotting, real-time PCR, and immune-fluorescence were performed using a one-way analysis of variance with Tukey's multiple comparison test. Patch clamp recording and temperature data were analyzed using paired *t*-tests. All statistical analyses were performed using GraphPad Prism 9.5.1 software (GraphPad Software, La Jolla, CA, USA). Data were considered significantly different when the *p*-value was <0.05. Results are presented as mean ± standard error of the mean (SEM).

## 3. Results

### 3.1. A subset of POMC neurons express the TRPA1 Channel in the ARC

**Figure 1.**
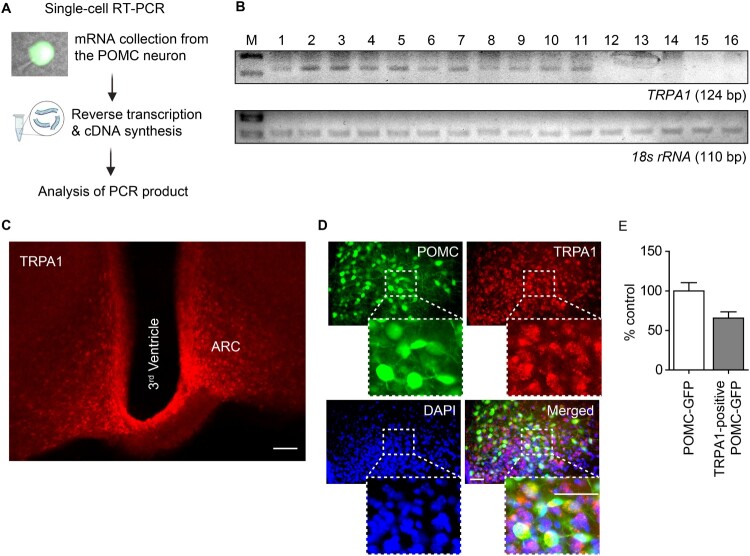
**Expression of TRPA1 mRNA and protein in ARC POMC neurons. (A)** Schematic diagram of single-cell RT-PCR analysis. **(B)** Representative gel electrophoresis images of TRPA1 mRNA and 18S rRNA expression. (**C**) Representative immunofluorescence images showing TRPA1 expression in the ARC (Scale bar 200 μm). (**D**) Colocalization of TRPA1 channels (red) with DAPI (blue) in POMC neurons (green) (Scale bar 50 μm). **(E)** Percentile analysis of the TRPA1-positive POMC neurons in the ARC. **Abbreviations:** TRPA1: Transient Receptor Potential Ankyrin 1; mRNA: messenger RNA; ARC: Arcuate Nucleus; POMC: Pro-opiomelanocortin; RT-PCR: Reverse Transcriptase Polymerase Chain Reaction; rRNA: ribosomal RNA; DAPI: 4′,6-diamidino-2-phenylindole.

Previous studies have shown that TRPA1 is expressed in multiple brain regions, contributing to thermogenesis regulation and lipid metabolism (Jha et al. 2015; De Logu et al. 2017). To determine TRPA1 expression in ARC POMC neurons, single-cell RT–PCR and IHC were performed. As shown in Figure 1A and B, approximately 70% of POMC neurons expressed TRPA1 mRNA. Furthermore, IHC revealed that at least 60% of POMC neurons co-localized with TRPA1 channels (Figure 1C–E). These data suggest that TRPA1 activity in POMC neurons can regulate their function.

### 3.2. Regulation of POMC neuronal activity via TRPA1 Channels in the hypothalamus

**Figure 2.**
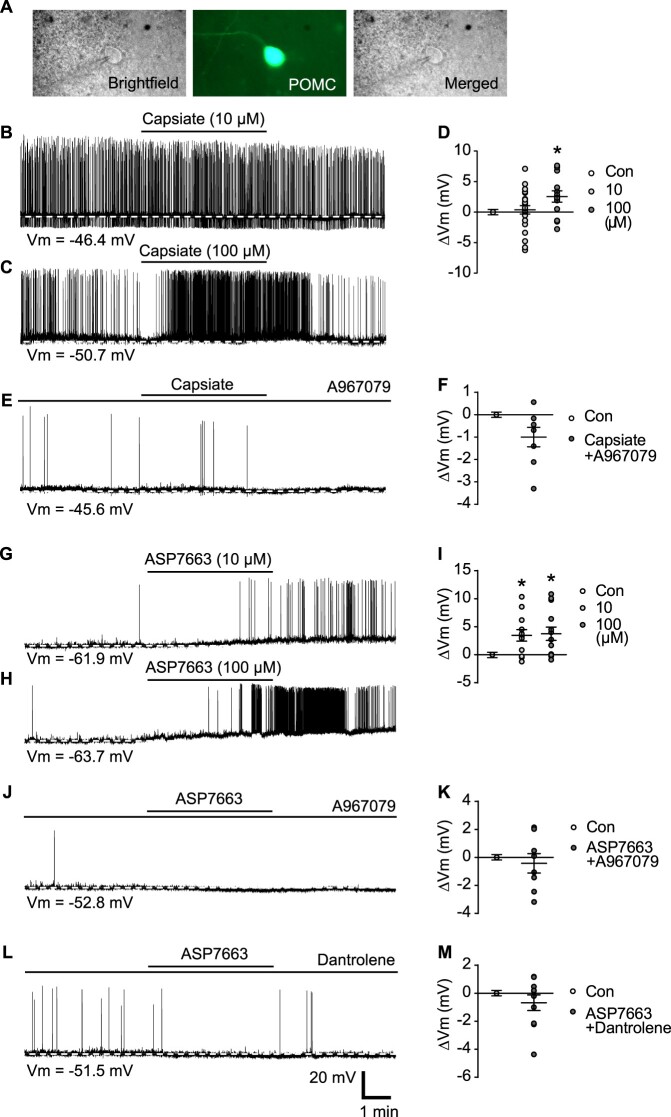
**Alteration of membrane potential in ARC POMC neurons in response to TRPA1 stimulation. (A)** Representative brightfield, infrared, and merged images illustrating whole-cell patch-clamp recordings in POMC neurons for capsiate treatment. **(B–C)** Representative traces of membrane potential changes in POMC neurons after 10 μM and 100 μM capsiate treatment. **(D)** Pooled data showing POMC neuron depolarization following 100 μM capsiate exposure. **(E)** Representative recording trace illustrating membrane potential responses to 100 μM capsiate with selective TRPA1 antagonist, A967079 (scale bar: 20 mV, 1 min). **(F)** Pooled data showing capsiate-induced POMC neuron depolarization with TRPA1 antagonist A967079. **(G–H)** Representative traces of membrane potential changes after 10 μM and 100 μM ASP7663 treatment. **(I)** Pooled data showing POMC neuron depolarization in response to 10 μM and 100 μM ASP7663 exposure. **(J)** Representative recording trace showing blockade of ASP7663-induced depolarization in POMC neurons by A967079. **(K)** Pooled data showing ASP7663-induced depolarization in the presence of TRPA1 antagonist A967079. **(L)** Representative recording trace showing blockade of ASP7663-induced depolarization in POMC neurons by dantrolene. **(M)** Pooled data showing ASP7663-induced depolarization with dantrolene. Data are presented as mean ± SEM. *p < 0.05 vs. control. **Abbreviations:** TRPA1: Transient Receptor Potential Ankyrin 1; ARC: Arcuate Nucleus; POMC: Pro-opiomelanocortin; SEM: Standard Error of Mean.

Several studies have demonstrated the role of TRP ion channels in regulating ARC POMC neurons (Jeong et al. 2018; Kelly and Wagner 2024). To investigate the role of TRPA1 in regulating POMC neuron activity, we performed patch-clamp recordings of POMC neurons treated with capsiate, a TRPA1 agonist derived from sweet pepper. Successful patch clamping was visualized by positioning the glass pipette in a POMC-GFP mouse brain slice, as shown in Figure 2A. In addition, comparative assessments using 10 and 100 µM capsiate determined the optimal concentration for activating POMC neurons (Figure 2B and C). Notably, treatment with 100 µM capsiate considerably depolarized POMC neurons (2.6 ± 0.9 mV, n = 13), whereas 10 µM capsiate had no significant effect (0.4 ± 0.7 mV, n = 23) (Figure 2B–D). To investigate whether capsiate-induced POMC depolarization is blocked by the selective TRPA1 antagonist, A967079 was used (Figure 2E and F). As shown in Figure 2F, A967079 effectively reduced the capsiate-induced depolarization (−1.0 ± 0.7 mV, n = 8), suggesting that TRPA1 plays an essential role in regulating POMC neuronal activity.

**Figure 3.**
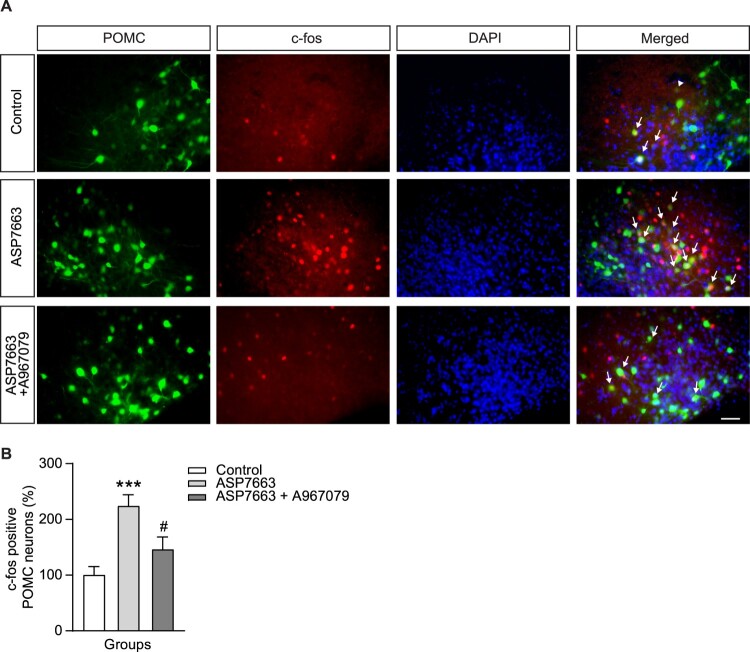
**Changes of c-fos expression in ARC POMC neurons after TRPA1 agonist and antagonist treatment. (A)** Immunohistochemical analysis showing c-fos expression in a subset of ARC POMC neurons following i.c.v. injection of ASP7663 (10 μM/μL). **(B)** Quantitative analysis of c-fos-positive POMC neurons in the ARC in each group; White arrows indicate POMC-eGFP neurons colocalized with c-fos in each group. Data are shown as mean ± SEM. ***p < 0.001 vs. control; ^#^*p* < 0.05 vs. ASP7663. Scale bar: 200 μm. **Abbreviations:** TRPA1: Transient Receptor Potential Ankyrin 1; ARC: Arcuate Nucleus; POMC: Pro-opiomelanocortin; SEM: Standard Error of Mean.

Capsiate effectively activates POMC neurons; however, its short half-life limits its applicability (Iida et al. 2003). Therefore, we replaced it with ASP7663, a selective TRPA1 agonist. Whole-cell patch-clamp recordings revealed that ASP7663 application caused membrane depolarization in POMC neurons (Figure 2G–I). ASP7663 substantially depolarized ARC POMC neurons at both 10 and 100 μM concentrations (10 μM: 3.5 ± 1.0 mV, n = 12; 100 μM: 3.7 ± 1.2 mV n = 13) compared to the control group (Figure 2G–I). These results suggest that ASP7663 effectively activates ARC POMC neurons. Moreover, A967079 treatment considerably suppressed the ASP7663-induced depolarization of POMC neurons (−0.4 ± 0.7 mV, n = 8), as shown in Figure 2J and K. Notably, TRPA1 is involved in intracellular calcium signaling (Shang et al. 2016; Talavera et al. 2020). As shown in Figure 2L and M, dantrolene, an inhibitor of intracellular Ca^2^⁺ release, markedly inhibited ASP-induced depolarization of POMC neurons (−0.7 ± 0.6 mV, n = 10). These data suggest TRPA1 regulates the activity of POMC neurons not only at the cell membrane but also through intracellular calcium signaling. To suggest solid evidence that TRPA1 regulates POMC neuronal activity, changes in c-fos expression, a marker of neuronal activity, were measured by IHC (Figure 3A). Following i.c.v. injection of ASP7663 (10 µM) c-fos expression levels increased in ARC POMC neurons. In addition, the ASP7663-induced increase in c-fos expression in POMC neurons was completely blocked by the TRPA1 antagonist A967079 (Figure 3A and B). These data suggest that TRPA1 is involved in regulating ARC POMC neuronal activity.

### 3.3. ARC POMC neurons establish neural connectivity with BAT

**Figure 4.**
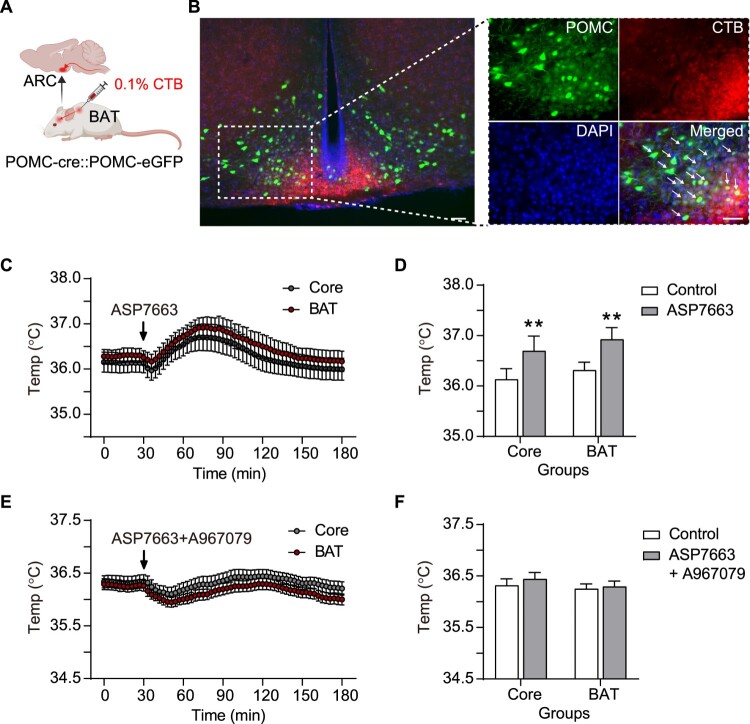
**POMC^TRPA1^-mediated modulation of BAT and core body temperatures. (A)** Schematic illustration of the retrograde tracer CTB administration into interscapular BAT. **(B)** Left panel: Merged image showing POMC distribution and CTB-positive area in the ARC of the hypothalamus. Right panel: Colocalization of CTB (red) with POMC neurons (green) and DAPI (blue) in the ARC. **(C)** BAT and core body temperature measurements following i.c.v. injection of the ASP7663 (n = 8). **(D)** Quantitative analysis of ASP7663 effect on BAT and core body temperatures. **(E)** BAT and core body temperature measurement following co-administration of A967079 and ASP7663 (n = 9). **(F)** Quantitative analysis of A967079 and ASP7663 co-treatment’s effect on BAT and core body temperatures. Data are shown as mean ± SEM. *p < 0.05, **p < 0.01 vs. control. **Abbreviations:** SEM: Standard Error of Mean; BAT: Brown Adipose Tissue; ARC: Arcuate Nucleus; POMC: Pro-opiomelanocortin; CTB: Cholera Toxin Subunit B; DAPI: 4′,6-diamidino-2-phenylindole.

Previous studies have demonstrated that hypothalamic POMC neurons project to peripheral organs through sympathetic innervation (Morelli et al. 2013; Jha et al. 2015; Wang et al. 2024). In the present study, we investigated whether the ARC POMC neurons innervate the interscapular BAT using retrograde neuronal tracing with mcherry-conjugated CTB (Figure 4A and B). After injecting 5uL of 0.1% CTB bilaterally into the BAT, mcherry expression was confirmed in the hypothalamus after 14 days. Following the bilateral administration of CTB into the BAT of POMC-cre::POMC-eGFP mice, neural tracing revealed that a subset of CTB-labeled ARC POMC neurons exhibited mcherry fluorescence, suggesting direct synaptic projections to the BAT (Figure 4B). This approach enabled the identification of direct synaptic connections between ARC POMC neurons and BAT, providing insights into the neural circuitry involved in the hypothalamic regulation of BAT thermogenesis.

### 3.4. Changes in BAT and body core temperature by POMC^TRPA1^

Considering that POMC neuronal activity is regulated by TRPA1 and that POMC neurons are neurally connected to BAT, we investigated whether POMC TRPA1 activity could regulate BAT thermogenesis. As shown in Figure 4C–D, i.c.v. injection of ASP7663, considerably elevated both BAT temperature (from 36.31 ± 0.1 °C to 36.93 ± 0.1 °C, n = 8) and core body temperature (from 36.13 ± 0.1 °C to 36.7 ± 0.1 °C, n = 8). Moreover, the ASP7663-induced increase in BAT temperature (from 36.25 ± 0.1 °C to 36.29 ± 0.1 °C, n = 9) and core body temperature (from 36.32 ± 0.1 °C to 36.44 ± 0.2 °C, n = 9) were blocked by A967079 (Figure 4E–F). These results demonstrate that POMC^TRPA1^ neurons can induce thermogenesis in BAT and core, suggesting that POMC^TRPA1^ may be a potential therapeutic target for promoting energy expenditure via thermogenesis.

### 3.5. Changes in the expression of thermogenic and lipid metabolism markers in BAT induced by POMC^TRPA1^

**Figure 5.**
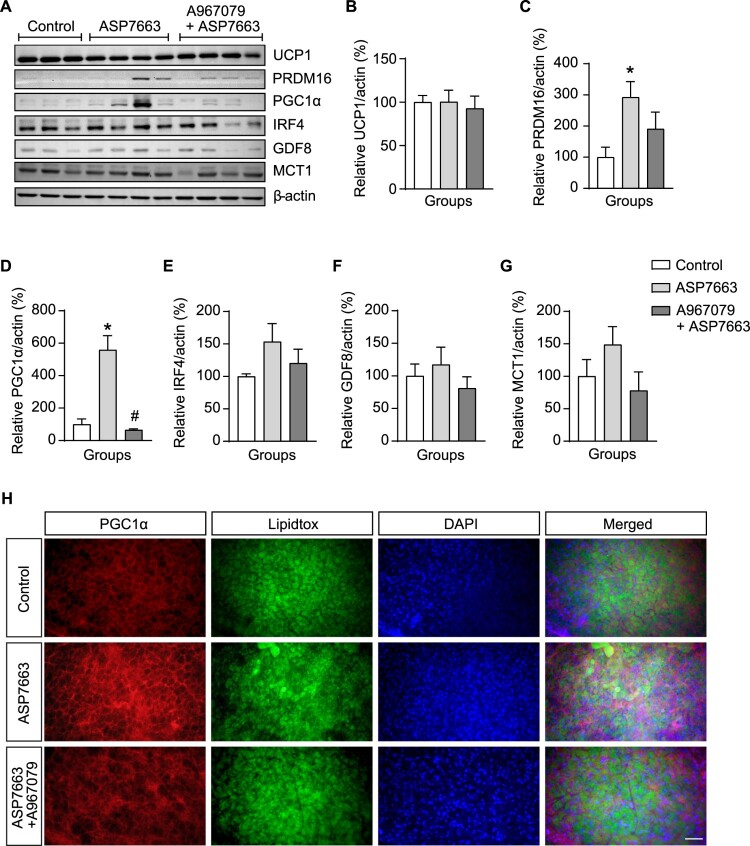
**POMC^TRPA1^-mediated modulation of thermogenesis makers in the BAT. (A)** Representative images of BAT thermogenic markers alteration in control, ASP7663, and A967079 + ASP7663 treated group. (**B–G**) Quantitative analyses of UCP1, PRDM16, PGC1α, IRF4, GDF8, MCT1 expression in the BAT (n = 7 each group), respectively. **(H)** Fluorescence images of BAT showing triple immunostaining for PGC1α (red), neutral lipids visualized with LipidTox (green), and DAPI (blue). Data are shown as mean ± SEM. *p < 0.05 vs. control, ^#^*p* < 0.05 vs. ASP7663. **Abbreviations:** BAT: Brown Adipose Tissue; POMC: Pro-opiomelanocortin; UCP1: Uncoupling protein 1; PRDM16: PR domain containing 16; PGC1α: peroxisome proliferator-activated receptor-gamma coactivator 1-alpha; GDF8: growth differentiation factor-8; MCT1: monocarboxylate transporter 1.

**Figure 6.**
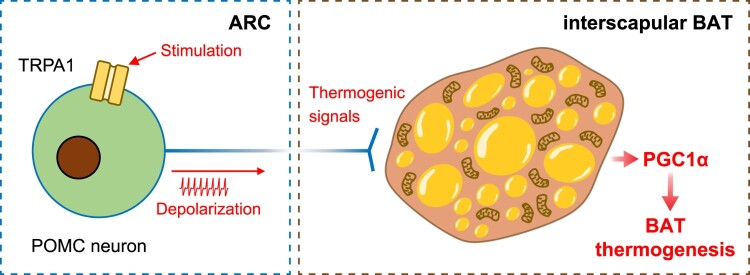
Schematic illustration depicting TRPA1 stimulation in ARC POMC neurons promoting BAT thermogenesis through the induction of PGC1α expression in BAT. **Abbreviations:** TRPA1: Transient Receptor Potential Ankyrin 1; ARC: Arcuate Nucleus; POMC: Pro-opiomelanocortin; PGC1α: peroxisome proliferator-activated receptor-gamma coactivator 1-alpha; BAT: Brown adipose tissue.

The BAT has an energy-burning function that utilizes fat to generate heat through non-shivering thermogenesis, facilitated by mitochondrial UCP1 expression (Scherer et al. 2011; Lasar et al. 2013; Uchida et al. 2017). Therefore, we monitored thermogenic marker expression in BAT after modulating POMC^TRPA1^ activity. As shown in Figure 5, POMC^TRPA1^ stimulation increased PRDM16 and PGC1α expressions (Figure 5C–D), but not IRF4, GDF8, and MCT1 (Figure 5E–G). Pretreatment with TRPA1 antagonist attenuated only PGC1α expression, which was increased by ASP7663 (Figure 5D and H). These results demonstrate that PGC1α plays a crucial role in BAT thermogenesis induced by acute stimulation of ARC POMC^TRPA1^ neurons (Figure 6).

## 4. Discussion

This study demonstrates that TRPA1 receptors expressed in hypothalamic POMC neurons regulate neuronal activation. In addition, hypothalamic POMC neurons are neurally connected to BAT, and POMC^TRPA1^ activation contributes to BAT thermogenesis. Notably, POMC^TRPA1^ stimulation modulates key thermogenic marker expression, including PGC1α and PRDM16. Collectively, these findings indicate that TRPA1-mediated activation of hypothalamic POMC neurons regulates BAT thermogenesis and promotes energy expenditure.

Hypothalamic POMC neurons are well-established regulators of energy balance, suppressing food intake and enhancing energy expenditure (Jeong et al. 2018; Tran et al. 2022). Previous studies have demonstrated that activating hypothalamic POMC neurons via TRPV1 channels enhances energy expenditure and alleviates metabolic impairments (Derbenev and Zsombok 2016; Jeong et al. 2018). Notably, TRPV1 and TRPA1 share structural similarities and often respond to the same pharmacological compounds. For instance, oleuropein aglycone, a dual agonist of TRPA1 and TRPV1, has been shown to enhance UCP1 expression in BAT and promote thermogenesis by increasing norepinephrine (NE) secretion (Oi-Kano et al. 2017). Although these findings indicate a potential role for TRPA1 in BAT thermogenesis, its involvement in hypothalamic POMC neurons remains unclear.

Various TRP channels stimulate POMC neuronal activity and influence energy expenditure. For instance, TRPC5 and TRPV1 regulate hypothalamic POMC neurons excitability within the energy expenditure-promoting mechanism (Götz et al. 2017; Jeong et al. 2018; Kelly and Wagner 2024). Moreover, TRPV1 agonist-induced BAT activation involving TRPA1- and TRPM8-expressing sensory nerves enhances thermogenesis and energy expenditure in mice (Iwasaki et al. 2008; Uchida et al. 2017; Haque and Ansari 2018). Consistent with these findings, Legrand et al. (2020) demonstrated that cumin aldehyde-induced TRPA1 activation exerts an anti-diabetic effect, which is associated with enhanced energy expenditure and increased fat oxidation. In addition, capsiate, a known TRPA1 agonist, increases Ca^2^⁺ influx in both channels and promotes thermogenesis and lipid metabolism (Uchida et al. 2017; Talavera et al. 2020). As shown in Figure 1, we first confirmed the expression of TRPA1 channels in hypothalamic POMC neurons. Furthermore, as shown in Figure 2, selective TRPA1 agonists effectively depolarized hypothalamic POMC neurons, and this depolarization was inhibited by selective TRPA1 inhibitors. These findings indicate that TRPA1 is expressed in hypothalamic POMC neurons and plays a crucial role in regulating their excitability.

In addition, capsiate induced a rapid but transient increase in neuronal firing, which may be due to its rapid metabolism and faster desensitization of the TRPA1 channel (Iida et al. 2003; Akopian et al. 2007; Ludy et al. 2011). In contrast, ASP7663 evoked a delayed but sustained response, even after washout (2G–H). This may be attributed to ligand-specific channel kinetics, such as slower desensitization and more sustained receptor activity compared to capsiate. Additionally, the persistent effect of ASP7663 was abolished when intracellular calcium signaling was inhibited (Figure 2L), suggesting that both TRPA1-mediated cation influx and intracellular calcium-dependent pathways synergistically contribute to the activation of POMC neurons. Consistent with previous reports (Shang et al. 2016), these results support the idea that TRPA1 function is not only influenced by the properties of the ligand, but also affected and modulated by intracellular calcium signaling pathways.

Hypothalamic POMC neurons are essential regulators of energy homeostasis, integrating peripheral metabolic signals and modulating sympathetic nervous system activity (Tran et al. 2022). Upon activation, POMC neurons release α-MSH, which binds to MC4R in the PVN and downstream sympathetic premotor neurons in the rostral medullary raphe, enhancing sympathetic outflow to BAT (Contreras et al. 2017). This activation leads to increased NE release from sympathetic nerve terminals, stimulating β_3_-AR in BAT and subsequently activating UCP1-mediated thermogenesis (Lowell and Flier 1997; Paulo et al. 2018). We employed CTB, a retrograde neuronal tracer, to identify neural connections between hypothalamic POMC neurons and BAT (Figure 4A–B). These connections suggest a potential role for TRPA1, a regulator of POMC neuronal activity, in BAT thermogenesis. Dysregulation of POMC neuronal activity or impaired sympathetic nervous system (SNS) signaling can lead to metabolic disorders, including obesity and thermogenic dysfunction. Recent studies have shown that genetic or pharmacological activation of POMC neurons enhances BAT thermogenesis, whereas inhibition of POMC activity reduces SNS-mediated BAT activation (Schneeberger et al. 2013; Labbé et al. 2015; Contreras et al. 2017).

Administration of ASP7663 activated ARC POMC neurons, leading to an increase in both BAT and core body temperatures (Figure 4C) and the expression of thermogenic markers, particularly PGC1α, whereas UCP1 expression remained unaffected (Figure 5B and D). PGC1α plays a key role in regulating mitochondrial transcriptional levels (Schlein et al. 2021; Ito et al. 2024). Additionally, PGC1α is a crucial regulator of BAT thermogenesis, as its depletion impairs BAT function (Gill and La Merrill 2017; Uchida et al. 2017; Abu Shelbayeh et al. 2023). As shown in Figure 5, acute stimulation of POMC neurons by a TRPA1 agonist increased PGC1α and PRDM16 expressions, key markers of BAT thermogenesis, whereas UCP1 expression remained unchanged. However, BAT thermogenesis induced by acute POMC^TRPA1^ activation was transient, with maximum change at 90 min (Figure 4C). This relatively short duration suggests that BAT thermogenesis may be regulated by PGC1α and PRDM16 rather than UCP1. As a master regulator of UCP1 expression, PGC1α is a key transcriptional coactivator governing BAT thermogenesis by interacting with transcription factors involved in thermogenic gene regulation (Uldry et al. 2006; Sharma et al. 2014). Further, PRDM16 induces a strong BAT phenotype by promoting PGC1α and UCP1 expressions (Seale et al. 2007). Additionally, changes in UCP1 expression induced by β_3_-AR agonists require at least 1–4 days (Galmozzi et al. 2014). These findings suggest that PGC1α and PRDM16 may serve as reliable indicators for evaluating BAT thermogenesis induced by acute stimulation of hypothalamic POMC^TRPA1^.

Our study highlights the important role of hypothalamic ARC POMC^TRPA1^ in maintaining energy homeostasis. Activation of POMC^TRPA1^ in the hypothalamus may contribute to a potential therapeutic strategy for combating obesity and diabetes by enhancing energy expenditure through BAT thermogenesis and increasing thermogenic markers expression.

## Authors’ contributions

Conceptualization, Halanobis A and Yang JH, Kim DH and Lee DK; methodology, Halanobis A, Yang JH, Byeon EH, Yang J and Kim W; validation, Kim DR, Park SW and Kim HJ; formal analysis, Kang DW, Halanobis A, Yang JH, Lee DK; investigation, Halanobis A, Yang JH, Lee DK; Funding acquisition, Kim DH, Lee DK, Park SW and Kim HJ; Drafting and revising of manuscript: Halanobis A and Yang JH, Kim DH and Lee DK. All authors have read and agreed to the published version of the manuscript.

## Supplementary Material

Supplemental Material

## Data Availability

All materials and data newly created in this work are available upon request.
