# Supplementary material for: Regulation of BAT thermogenesis via TRPA1-expressing hypothalamic POMC neurons
Source: Anim Cells Syst (Seoul). 2025 Sep 29;29(1):584–97. doi: 10.1080/19768354.2025.2559611 (PMC12486466; doi:10.1080/19768354.2025.2559611)
Supplement: Supplemental Material [file TACS_A_2559611_SM2963.docx]

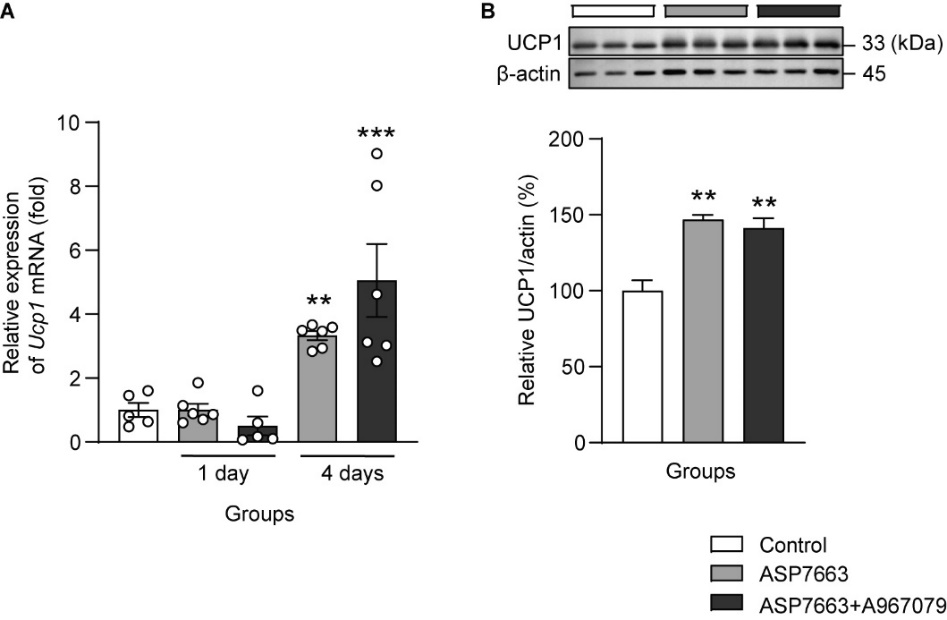


Fig. R1. TRPA1 agonist ASP7663 increases Ucp1 mRNA and protein expression, which is not inhibited by the TRPA1 antagonist A967079. (A) Relative mRNA expression of *Ucp1* BAT following treatment with ASP7663 alone or in combination with A967079 for 1 or 4 days (n=5~6). *Ucp1* mRNA levels were significantly increased after 4 days of ASP7663 treatment, and this effect was maintained even with co-treatment with A967079. (B) Western blot analysis and quantification of UCP1 protein expression in BAT after 4 days of treatment. UCP1 levels were elevated in both ASP7663 and ASP7663+A967079 groups compared to control (n=3). Data are presented as mean ± SEM. **p < 0.01, ***p < 0.001 vs. control.
